# The Androgen Receptor Bridges Stem Cell-Associated Signaling Nodes in Prostate Stem Cells

**DOI:** 10.1155/2016/4829602

**Published:** 2016-01-10

**Authors:** Alastair H. Davies, Amina Zoubeidi

**Affiliations:** ^1^Vancouver Prostate Centre, Vancouver, BC, Canada V6H 3Z6; ^2^Department of Urologic Sciences, Faculty of Medicine, University of British Columbia, Vancouver, BC, Canada V6H 3Z6

## Abstract

The therapeutic potential of stem cells relies on dissecting the complex signaling networks that are thought to regulate their pluripotency and self-renewal. Until recently, attention has focused almost exclusively on a small set of “core” transcription factors for maintaining the stem cell state. It is now clear that stem cell regulatory networks are far more complex. In this review, we examine the role of the androgen receptor (AR) in coordinating interactions between signaling nodes that govern the balance of cell fate decisions in prostate stem cells.

## 1. Introduction

Stem cells, characterized by their ability to self-renew (divide and create additional stem cells) and generate differentiated functional cell types, have been derived from the embryo as well as various adult organs [1]. It is customary to classify stem cells into two major types according to their developmental potential: embryonic stem cells (ESCs) and somatic stem cells. ESCs are pluripotent, self-renewing cells localized to the inner cell mass of the developing blastocyst that are capable of generating all cell types of the body. As development proceeds, pluripotent ESCs disappear as more restricted (multipotent) somatic stem cells, such as haematopoietic stem cells and neural stem cells, that can only give rise to cell types within a particular lineage. Although the privilege of differentiating into any of the hundreds of cell types in the human body is reserved for the ESCs, adult somatic stem cells residing within an organ or tissue nevertheless retain some characteristics of their early ESC counterparts, including the capacity to self-renew while keeping their repertoire of differentiation programs on hold.

Deciphering the regulatory circuitry underlying stem cell pluripotency and self-renewal is an important key to understanding both normal and, in the case of cancer, abnormal development. Here, we review the recent advances that demonstrate the presence and involvement of the androgen receptor (AR) in both normal stem cells and cancer stem cells (CSCs), particularly those associated with the prostate. We will discuss how the AR fits into the molecular circuitry that maintains the pluripotent and self-renewal state. The role of the stem cell niche in regulating the AR will be analyzed, together with the clinical implications.

## 2. The AR as a Regulator of the Stem Cell State

The AR is a ligand-inducible transcription factor that in response to androgens (namely, testosterone and 5*α*-dihydrotestosterone) dimerizes, translocates to the nucleus, and binds to androgen response elements (AREs) in the promoter region of target genes. Subsequent interaction with cofactors allows the AR complex to stimulate or inhibit gene transcription [2]. The AR is expressed in various tissues [3] and has been linked to several diseases, most notably prostate cancer [4]. While classically viewed as a driver of cell growth and survival, emerging evidence suggests that stem cells can be influenced by androgen and AR signals both* in vitro* and* in vivo* (Table 1). Thus, the AR may serve a currently underappreciated role in shaping the properties and defining the potential of stem cells.

### 2.1. AR in Embryonic Stem Cells

Within the last decade several advances have made it possible to recapitulate in ESC cultures the key events that regulate lineage commitment in the embryo [5]. Manipulation of the AR axis in ESCs revealed that it acts as a negative regulator of the stem cell state. As ESCs begin to differentiate, AR levels rise in a stage-dependent manner [6], suggesting that it may be functioning to suppress the stem cell phenotype. This hypothesis was confirmed by treating ESC cultures with steroid hormones* in vitro*. In particular, testosterone treatment efficiently stimulated ESC differentiation into cardiac mesoderm and, in turn, functional cardiomyocytes [7, 8]. Conversely, treating ESCs with nilutamide, an antiandrogen that inhibits AR activity, increased proliferation and enhanced self-renewal capacity [9].

### 2.2. AR in Prostate Stem Cells

Although cell diversification is largely complete at birth, organs must possess a mechanism to replenish cells as they die, by either normal wear and tear (homeostasis) or injury. To accomplish this feat, many developing organs set aside life-long reservoirs of somatic stem cells that retain some of the versatile characteristics of ESCs, such as capacity for self-renewal.

The classic androgen ablation and replacement experiments demonstrated that the prostate epithelia possess extensive regenerative capacity thus providing the earliest evidence for the existence of prostate stem cells [10]. More recent studies, utilizing specific cell surface markers and genetic lineage tracing approaches, have produced direct evidence for prostate stem cells. Correlative and* in vitro* experiments infer that prostate stem cells reside within the basal cell layer as basal cells not only are slow cycling and express many stem cell associated genes such as telomerase, bcl-2, and p63, but also have low level of the AR [11–13]. On the other hand, we recently learned from the study of Wang et al. a small subset of luminal cells that survive castration (termed CARNs for castration-resistant Nkx3.1-expressing cells) can self-renew* in vivo* and regenerate a prostate in renal grafts [14]. It is important to note that despite a luminal phenotype, the origin of CARN cells is unknown and it is possible that basal cells adapt a CARN cell phenotype in castrated mice.

Despite these complexities, the overwhelming consensus is that prostate stem cells have a basal origin. For instance, prospectively purified Lin−/Sca-1+/CD49f+ basal cells can establish spheres and colonies* in vitro* as well as regenerate prostate ducts in renal grafts [15]. Notably, the expression of the AR was found to be very low in these cells. In another study, a single Lin−/Sca-1+/CD133+/CD44+/CD117+ basal cell was capable of reconstituting a prostate in the kidney capsule of recipient mice [16]. Garraway et al. demonstrate that a small population of human prostate cells with a basal phenotype and low AR expression is sufficient to induce prostatic gland structures* in vivo* [17]. Finally, elegant lineage-marking experiments identified a population of AR-negative basal multipotent stem cells with the capacity to differentiate into each of the prostate epithelial lineages (basal, luminal, and neuroendocrine cells) [18]. Thus, it can be concluded that prostate stem cells are most likely AR-negative.

## 3. The AR in Prostate Cancer Stem Cells

There is increasingly awareness that deregulated “stem cells” may be the real culprit for cancer growth, dissemination, and therapy resistance [19–21]. Colloquially referred to as cancer stem cells it is not yet understood if these cells are the progeny of mutated somatic stem cells [22–25] or if they arise* de novo* from reactivation of stem cell transcriptional networks in more differentiated cell types [26–28]. Irrespective of their origin, parallels can be drawn between somatic stem cells and CSCs. Both types of cells self-renew, although somatic stem cells do so in a highly regulated manner while CSCs are more poorly controlled. Moreover, both types of cells differentiate; somatic stem cells generate normal, mature cells whereas CSCs generate phenotypically diverse nontumorigenic cancer cells [20]. The phenotypic similarity between normal and cancer stem cells raises the possibility that CSCs are “diseased” stem cells and thus targeting stem cell-associated signaling nodes may represent a rational strategy to improve cancer therapy.

### 3.1. The Origin of Prostate Cancer Stem Cells

It has been suggested that normal stem cells acquire genetic and/or epigenetic alterations to transform into CSCs. In particular, the fact that prostate cancer stem cells (CD44+/CD133+/*α*2*β*1+) share antigenic properties with prostate stem cells (CD133+/*α*2*β*1+) supports the idea that they arise from the normal stem/progenitor cell counterpart [29–31]. This notion is further exemplified by the fact that CSCs isolated from human prostate tumors express basal markers (such as p63), but not the AR or markers of luminal differentiation [32], mirroring the phenotype of normal prostate stem cells. However, this dogma has recently been challenged by the discovery that differentiated, postmitotic cells have the capacity to ascend the tumor hierarchy and reenter the CSC state. The study of Gupta et al. showed us that breast cancer cell populations can interconvert between phenotypic states [26]. In other words, CSCs can arise* de novo* from non-stem cells. A similar phenomenon has been described in prostate cancer whereby cells can dedifferentiate to a CSC state under the pressure of stressors, such as chemotherapy or nutrient deprivation [33].

### 3.2. Prostate Cancer Stem Cells Have Low AR Expression and/or Activity

A series of seminal papers published in 2005 provided the earliest evidence for the existence of CSCs in prostate cancer: first, a population of tumorigenic cells with high expression of the ABCG2 drug efflux pump (termed the side population) was isolated in human prostate cancer LAPC9 xenografts [34]; second, ABCG2 was discovered to mediate the efflux of androgen in putative CSCs [35]; and third, cells isolated from prostate tumors based on the cell surface markers CD44+/CD133+/*α*2*β*1+ could self-renew and differentiate to generate a phenotypically mixed population [29]. Since then, prostate CSCs have been isolated and purified via expression of cell surface markers, including CD44 [36], CD133, CXCR4 [37], and TRA-1-60/CD151/CD166 [38]. Notably, all of the identified subsets of putative prostate CSCs lack AR expression or have low AR activity, which suggests that these cells, like normal prostate stem cells, are inhibited by AR signaling.

Indeed, knockdown of the AR in immortalized epithelial cells results in the expansion of the CSC pool [39], while overexpression of the AR in CD133-positive CSCs reduces their self-renewal capacity [40]. Keeping with these findings, the CSC population is expanded dramatically following androgen deprivation therapy (ADT) both in mouse models and in patient tumors [40–42]. For example, Qin and colleagues discovered a small population of cells defined by low expression of prostate specific antigen (PSA), a transcriptional target of the AR, that resist castration and exhibit heightened self-renewal capacity [43]. These cells are also capable of asymmetric cell division to regenerate a phenotypically mixed tumor, including AR-positive and PSA-positive cells. Notably, the ALDH+/CD44+/*α*2*β*1+ CSC population can be prospectively purified in PSA−/lo cells indicating that low AR expression and/or activity is a distinguishing feature of CSCs, at least in the prostate.

### 3.3. Low AR Expression Is a Common Feature of Cancer Stem Cells

Circumstantial evidence suggests that the AR may also play a role in leukemia, breast, and brain CSCs. In leukemia, AR expression is silenced, in part, due to increased methylation of CpG islands in the AR promoter [44]. Similarly, triple-negative breast cancer, which is enriched in CSCs, is generally AR-negative and these tumors are most likely to recur [45]. Finally, glioblastoma stem cells require STAT3 for proliferation and maintenance of multipotency [46], and loss of the AR yields STAT3 activation [47]. Although it appears that the AR may have a far broader role in regulating CSCs than initially imagined, clearly, further studies are required to evaluate AR regulation of CSC phenotype in other cancers outside of prostate cancer.

## 4. The AR in Stem Cell Signaling Networks

**Figure 1:**
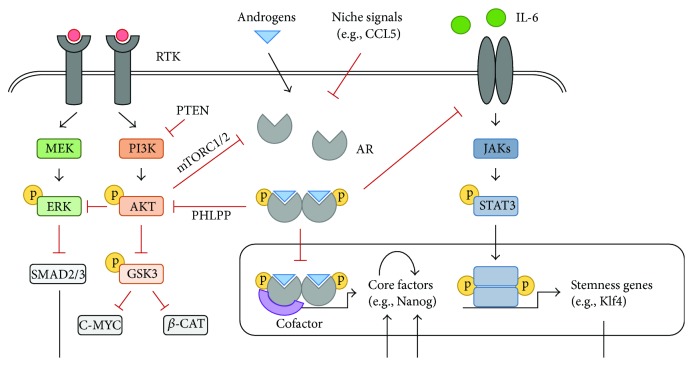
Regulation of interconnected stem cell signaling nodes by the AR. Activation of the AR negatively regulates the core pluripotency transcription factors (Nanog, Sox2, and Oct4) as well as signaling cascades that reinforce a robust stem cell state. The AR (1) acts as a transcriptional repressor at the Nanog and Sox2 promoters; (2) inhibits PI3K/Akt signaling through induction of PHLPP, which dephosphorylates Akt to facilitate ERK pathway activation leading to Nanog gene repression, as well as GSK3-mediated c-myc and *β*-catenin repression; and (3) blocks STAT3-mediated transcription of stem cell-associated genes by inhibiting IL-6. Suppression of the AR by factors in the stem cell niche, such as CCL5, relieves this inhibition to favor self-renewal and pluripotency over differentiation. AR, androgen receptor; *β*-CAT, *β*-catenin; CCL5, chemokine ligand 5; IL-6, interleukin-6; JAK, Janus kinase; PHLPP, PH domain and leucine rich protein phosphatase; RTK, receptor tyrosine kinase.

Complex regulatory networks are known to maintain cells in distinct cell fates [48, 49]. These developmental signaling pathways that govern pluripotency and self-renewal in normal stem cells are generally thought to be shared with cancer stem cells [50, 51]. For example, it was recently demonstrated that prostate tumors with a stem-like phenotype molecularly resemble normal stem cells residing within the human prostate [52]. Although the rewiring of stem cell regulatory networks remains poorly understood, emerging studies suggest that the AR coordinates the activity of stem cell-associated signaling nodes that “tip-the-balance” between a stem cell and differentiated cell state (Figure 1).

### 4.1. The Core Regulatory Circuitry

The pluripotent state is largely governed by the core transcription factors Oct4, Sox2, and Nanog [53, 54]. Oct4 and Nanog were first identified based on their relatively unique expression in ESCs and genetic knockout studies showing that they are essential for establishing or maintaining a robust pluripotent state [55–57]. Oct4 functions as a heterodimer with Sox2, thus placing it among the core regulators [58]. Notably, forced expression of Oct4 and Sox2 facilitates the reprogramming of somatic cells into induced pluripotent stem (iPS) cells [59]. Although ESCs can be propagated in the absence of Nanog, it cooccupies most sites with Oct4 and Sox2 throughout the ESC genome and functions to promote a stable undifferentiated ESC state [60, 61].

How might cells integrate signals from their environment and choose whether to remain pluripotent or differentiate into progenitors? The AR has been implicated as a “molecular switch” that functions in coordinately regulating the expression of the pluripotency transcription factors: Sox2, Oct4, and Nanog. In ESCs and prostate epithelial cells increased AR signaling decreases Sox2 expression. This is the result of AR binding to the enhancer element within the* Sox2* promoter where it acts as a transcriptional repressor [62]. The AR also binds directly to the* Nanog* promoter [63], and in cell lines and patient specimens Nanog and the AR are reciprocally expressed [64, 65]. Finally, while no studies have directly addressed the role of the AR in the regulation of Oct4, it has been reported that Oct4 is increased during neuroendocrine differentiation [66, 67] and neuroendocrine cells are largely AR-negative [68]. Taken together, these studies suggest that the AR functions upstream of Sox2, Oct4, and Nanog to orchestrate their coordinated regulation during cell fate transitions.

### 4.2. PI3K/Akt Signaling: Cross-Talk with Myc and ERK

Phosphoinositide-3 kinase (PI3K)/Akt has been implicated in regulating stem cell transcriptional networks given the discovery that ESCs lacking* Pten*, which encodes a phosphatase that antagonizes PI3K signaling, exhibit enhanced proliferation and form teratomas composed of undifferentiated cells* in vivo* [69]. Similarly, the deletion of* Pten* during haematopoiesis was found to increase the HSC pool [70]. These findings have been corroborated by studies in human ESC cultures where inhibition of PI3K using the small molecule inhibitor LY294002 or removal of PI3K activators contributes to loss of pluripotency and triggers differentiation [71–73]. Like normal stem cells, the proliferation as well as maintenance of CSCs is also dependent on PI3K/Akt signaling. Pten knockdown in DU145 prostate cancer cells yields an enrichment in CD133+/CD44+ CSCs, while treatment with LY294002 reduces sphere formation [74].

Recent studies indicate that androgen depletion or knockdown of AR expression results in elevated levels of activated Akt [75, 76], suggesting that AR negatively regulates PI3K/Akt signaling. Akt phosphorylates numerous target proteins, notably, GSK3 leading to its degradation. One effect of GSK3 inhibition in this context is to stabilize *β*-catenin, a transcriptional coactivator implicated in stem cell regulation [77]. In particular, it has been proposed that the AR inhibits the self-renewal of ESCs and BM-MSCs through suppression of Akt signaling [9, 78]. This is, at least in part, mediated via the AKT/GSK3/*β*-catenin signaling pathway as knockdown of *β*-catenin prevents BM-MSC differentiation in response to androgens [79].

AKT-mediated GSK3 inhibition also functions to stabilize c-myc [80], a key regulator in the maintenance of pluripotency and self-renewal. Interest in Myc function in stem cells was ignited by studies linking both c-myc and N-myc to the generation of iPS cells [59, 81], the regulation of self-renewal in ESCs [80, 82], and tumor stem cell maintenance [83]. Knockout of Myc in ESC lines yields a profound disruption in pluripotency and self-renewal, while inducing differentiation into ectoderm, mesoderm, and endoderm derivatives [84]. Genomic studies have suggested that c-myc targets are involved predominately in cellular metabolism, cell cycle, and protein synthesis pathways. In particular, Myc-centered transcriptional network composed of over 500 genes is upregulated in ESCs, iPS cells, and poorly differentiated, stem-like tumors emphasizing the pervasive nature of the Myc regulatory network in maintaining the stem cell state [85].

Apart from Myc, a second downstream target of the PI3K/Akt signaling cascade that supports a stem cell state is the core pluripotency factor Nanog. Nanog expression is downregulated following PI3K inhibition [86, 87] and this can be attributed to cross-talk between the PI3K/Akt and ERK signaling pathways. In particular, PI3K/Akt suppresses ERK signaling, which has a well-established role in antagonizing pluripotency in ESCs [88, 89]. This likely involves ERK-mediated phosphorylation of Smad proteins resulting in decreased activity and nuclear translocation [90]. Thus, under self-renewing conditions, the absence of ERK signaling permits Smad2/3 to transcriptionally activate Nanog in addition to other pluripotency-associated genes [73, 91]. A second feedback loop between the AR and ERK further dampens ERK activity in cells with low AR expression [92]. Together, these studies raise the possibility that negative regulation of PI3K/Akt by the AR potentiates Myc and/or Nanog expression to maintain pluripotency and self-renewal.

### 4.3. STAT3 Signaling Reinforces the Stem Cell State

A role for Signal Transducer and Activator of Transcription 3 (STAT3) in sustaining pluripotency and self-renewal capacity has been described in ESCs [93, 94] as well as CSCs, notably glioblastoma stem cells [46], breast cancer stem cells [95], and prostate cancer stem cells [47]. For instance, increasing STAT3 activity is sufficient to reprogram primed pluripotent stem cells back to naive-like ESCs [96], while expressing a dominate negative STAT3 mutant in ESCs abrogates self-renewal and promotes differentiation [94]. Likewise, treatment of DU145 prostate cancer cells with the STAT3 inhibitor galiellalactone reduces the frequency of ALDH-positive CSCs [97].

The STAT3 transcription factor is activated by the binding of inflammatory cytokines or growth factors in the interleukin- (IL-) 6 and IL-10 family to their cognate receptors on the cell surface. This potentiates intracellular signal transduction cascades in which receptor-associated Janus kinases (JAKs) phosphorylate STAT3 leading to its dimerization and nuclear translocation [98]. In particular, STAT3-mediated upregulation of Klf4 and/or Tfcp2L1 is sufficient to revert cells back to a pluripotent ESC-like state [99, 100]. A global analysis of promotor occupancy revealed Klf4 directly regulates a large, feed-forward loop that contains the core pluripotency factors and also occupies the c-myc promoter to potentiate a stem cell state [101]. The key role for STAT3 in maintaining pluripotency and self-renewal is supported by the observations that JAK/STAT3 signaling is a limiting factor in the reprogramming of somatic cells to naïve pluripotency [102], and that JAK/STAT3 signaling can be dominant over antagonistic FGF/ERK signaling to reinforce a pluripotent state [103].

Recent evidence suggests that STAT3 is negatively regulated by the AR. In prostate cancer cell lines downregulation of the AR increases STAT3 signaling, which is required for CSC maintenance. Moreover, in human prostate tumor tissue, cells with low AR expression exhibit high STAT3 activity and coexpress CSC markers, including Nanog and CD44 [47]. It has been proposed that AR downregulation increases IL-6 expression and, in turn, STAT3 activation. Notably, IL-6 has been implicated in the generation of iPS cells via activation of the JAK/STAT target Pim1 [104]. Inhibition of JAK/STAT3 signaling by gene silencing or using an IL-6 receptor fusion protein (IL-6^RFP^), which acts as a cytokine trap to sequester soluble IL-6, exhausts the CSC population in both murine and human prostate cancer models [47]. This is consistent with studies in glioblastoma showing that inhibition of IL-6 or its receptor, IL-6R*α*, diminishes STAT3 activation and, in turn, the number of glioblastoma stem cells [105].

### 4.4. Transcriptional Cofactors Reprogram the AR

The AR does not function in isolation, but rather associates with cofactors that can alter its function. In this way the AR can be “reprogrammed.” Of particular interest is enhancer of zeste homologue 2 (EZH2), an epigenetic regulator with a well-documented role in regulating cell fate [106]. A pioneering study by Xu and colleagues demonstrated that in androgen-independent prostate cancer EZH2 interacts with the AR, which alters the AR cistrome (genomic binding sites) to activate a unique transcriptional program [107]. While this study did not address EZH2 and AR interaction within the CSC population* per se*, it is tempting to speculate that noncanonical AR signaling may be involved in regulating the stem cell state.

## 5. AR Regulation by the Stem Cell Niche

The stem cell niche provides a microenvironment that is capable of protecting and perpetuating the self-renewing, undifferentiated state of stem cells. It is composed not only of stem cells but also of a rich array of neighbouring differentiated cell types which secrete a rich milieu of extracellular matrix and other soluble factors that allow stem cells to manifest their unique intrinsic properties [108].

It has been recently emerged that BM-MSCs recruited to tumors secrete the chemokine CCL5 (also called RANTES), which acts in a paracrine fashion to suppress AR signaling in neighbouring cancer cells [109]. This results in an increased CSC population, suggesting that BM-MSCs regulate the stem cell phenotype by secreting CCL5 [109, 110]. This phenotype is reversible and is dependent on CCL5-mediated upregulation of hypoxia inducible factor 2*α* (HIF2*α*), which alters AR:HSP90 interaction to suppress AR transactivation [111].

Similarly, endothelial cells secrete IL-6 to suppress the AR signaling axis [112]. This coincides with expansion of the stem cell pool, and treating mice with soluble IL-6 receptor fusion protein reduces the CSC population in prostate cancer [47]. Notably, endothelial cells have been reported to provide a niche for HSC expansion [113], supporting the notion that these cells promote the maintenance of stem cells that reside in close contact.

One final note is that cross-talk between non-stem and stem cell niches is not likely to be unidirectional. In fact, transcriptional profiling of stem cells reveals that they synthesize a number of growth factors that appear tailored for their neighbouring niche cells [114]. For example, in transwell assays BM-MSCs migrate avidly towards media derived from breast cancer cell lines [115]. Although the cellular communication pathways remain poorly defined, understanding how the stem cell niche is constructed will be a rich area for future studies.

## 6. Conclusions and Outlook

How do regulators of the stem cell gene expression program produce a self-renewing cell that remains poised for differentiation? Part of the answer is that the AR functions as a finely balanced “molecular switch,” integrating extrinsic signals to control the core pluripotency transcription factors as well as key signaling pathways that reinforce the stem cell state, such as PI3K/Akt and STAT3. In its “off” state the AR supports a transcriptional program that favors self-renewal, yet once activated the AR rewires the transcriptional circuitry to drive differentiation.

Overall, the AR pathway functions at critical crossroads in balancing stem cell self-renewal versus differentiation. Indeed, the importance of the AR in regulating stem cell plasticity is evident by its conserved role across embryonic and somatic stem cells, in addition to cancer stem cells. Future avenues of research will continue to advance our knowledge and understanding of the AR function in stem cells. These include, but are not limited to, determining how the AR directs differentiation along a specific lineage, ascertaining the role of AR cofactors, and understanding the influence of specific stem cell niches on AR expression. Such studies will lead to optimizations in stem cell-based therapy as well as new drug targets in CSCs to improve cancer outcomes.

## Figures and Tables

**Table 1:** The effect of androgens and/or AR expression on stem cell populations.

Stem cell type	Effect of AR/androgen	Effect of AR inhibition	References
Embryonic stem cell	Cardiomyocyte differentiation	Enhanced self-renewal Increased proliferation	[7–9]

Haematopoietic stem cell	Erythropoiesis Neutrophil differentiation	Expansion of MEPs Neutropenia	[116–119]

Mesenchymal stem cell	Reduced self-renewal Myogenic differentiation	Enhanced self-renewal Enhanced migratory ability	[120–123]
	Osteogenic differentiation		

Neural stem cell	Decreased proliferation	Increased proliferation	[124]

Prostate stem cell	Prostate epithelial differentiation	Enhanced self-renewal	[15, 31]

Prostate CSC	Reduced self-renewal	Enhanced self-renewal Expansion of CSC pool	[40, 43]

AR, androgen receptor; CSC, cancer stem cell; MEP, megakaryocyte-erythroid progenitor.
